# Elements behind sedentary lifestyles and unhealthy eating habits in individuals with severe obesity

**DOI:** 10.1080/17482631.2022.2056967

**Published:** 2022-03-31

**Authors:** Vicente J. Beltrán-Carrillo, Ángel Megías, David González-Cutre, Alejandro Jiménez-Loaisa

**Affiliations:** a Department of Sport Sciences, Sports Research Centre, Miguel Hernández University of Elche, Elche (Alicante), Spain; b Department of Didactics of Physical Education, Artistic and Music, Faculty of Education, University of Castilla-La Mancha, Toledo, Spain

**Keywords:** Physical activity, diet, comfort eating, bariatric surgery, qualitative

## Abstract

**Purpose:**

This study examines elements behind sedentary lifestyles and unhealthy eating habits in individuals who lived with severe obesity during many years of their lives.

**Methods:**

Ten post-bariatric surgery patients participated in semi-structured interviews 7 months after surgery. A year later, a second round of interviews was also conducted to address some knowledge gaps related to the study purpose. The qualitative data collected were analysed through content analysis.

**Results:**

Embarrassment about showing their body publicly, pain and poor physical condition, and bad experiences in non-inclusive physical activity contexts were found as potential elements that contributed to their adherence to sedentary lifestyles. Poor family food education, loss of a loved one, family problems, arguments or disputes, and past traumatic events (e.g., childhood sexual abuses) could be related to their development of unhealthy eating behaviours. Findings also pointed out that participants’ failed attempts to lose weight provoked them anxiety, feelings of failure and rebellion, and influenced their return to an unhealthy diet and a sedentary lifestyle.

**Conclusions:**

This study may be helpful to reveal some elements which could be related to the origin and perpetuation of severe obesity, and to design prevention/treatment strategies from a more holistic, sensitive, and respectful perspective.

## Introduction

Severe obesity, which is defined as having a body mass index (BMI) greater than or equal to 40 kg/m^2^, or a BMI greater than 35 kg/m^2^ with at least one serious obesity-related condition, is associated with an increased risk of suffering from certain non-communicable diseases such as cardiovascular ones, diabetes, osteoarthritis, or some cancers (World Health Organization, 2021). Current data suggest that the number of individuals in the heaviest BMI (>40 kg/m^2^ and >50 kg/m^2^) constitutes the fastest growing obesity subgroup in countries such as the USA (Ward et al., 2019) or Spain (Hernáez et al., 2019).

The main conditions that explain the aetiology of obesity are complex and manifold, and usually lie in a blend of behavioural, environmental, sociocultural, and psychological elements, along with genetic or biological attributes. Given that a long-term energy imbalance between expended and consumed calories is the main driver of obesity (Romieu et al., 2017), academics have frequently supported an obesity discourse which associates the responsibility for obesity with individual behaviour (Gard, 2011; Puhl & Heuer, 2010). This discourse focuses on physical inactivity and food consumption as the root elements of increased rates of obesity, blaming and stigmatizing individuals for their own obesity to press them for behaviour change (Gard, 2011), and therefore fostering psychological distress and emotional imbalances (Puhl et al., 2020).

However, inactivity and food consumption are associated with diverse environmental, cultural, and psychosocial elements as well. In fact, the fundamental reasons of obesity are often societal, resulting from a “toxic environment” that promotes physical inactivity and the consumption of calorie-dense foods (Brownell & Horgen, 2004). For instance, authors such as Rolls et al. (2007) and Ruhlman (2017) have pointed out the way in which the abundance and variety of products offered by the current food market can lead individuals to overeat. Other elements, such as food insecurity or prolonged stress that socio-economically disadvantaged people often experience, have also been related to the acquisition and perpetuation of obesity (Distel et al., 2019; Konttinen et al., 2013; Scott et al., 2012).

Given that obesity is a highly complex phenomenon, research should analyse it from different perspectives and methods. If academics and health professionals want to achieve a deeper understanding of obesity, and severe obesity in particular, it would be desirable to include the subjective perceptions of this population. Qualitative methodology gains therefore importance. In fact, some qualitative studies have already explored the subjective perceptions of people with obesity concerning the genealogy and course of their condition (Braun et al., 2014; Brogan & Hevey, 2009; Haga et al., 2020; Toft & Uhrenfeldt, 2015; Ueland et al., 2019).

Qualitative research shows that individuals experience obesity as something that has always been present (Braun et al., 2014) and describe personal stories of complex struggles of short-term weight loss and longer-term weight gain, usually characterized by a sense of failure (Greener et al., 2010; Haga et al., 2020; Toft & Uhrenfeldt, 2015). However, severe obesity could be related to early childhood experiences as lack of affection, loss of significant people, or parents’ divorce (Ueland et al., 2019). Other studies have shown that food is often used as a coping mechanism by people with higher weight, particularly when they are sad, anxious, stressed, lonely, or frustrated (Brogan & Hevey, 2009; Puhl & Heuer, 2010; Ueland et al., 2019). Bidirectional associations between depression and obesity have also been found (Chauvet-Gelinier et al., 2019; Luppino et al., 2010). Moreover, some specific psychological problems of obesity have been described, like binge eating disorder or night-eating syndrome, both highly associated with severe obesity (McCuen-Wurst et al., 2018).

Sedentary lifestyles also contribute to obesity, and individuals with severe obesity report different barriers to physical activity (PA). Common non-obesity-related barriers to PA include lack of self-efficacy, safety, motivation, or knowledge to engage in PA (Napolitano et al., 2011; Toft & Uhrenfeldt, 2015; Zabatiero et al., 2018), long workdays, traffic and darkness, family commitments, limited financial resources or unfavourable weather conditions (Beltrán-Carrillo et al., 2019; Toft & Uhrenfeldt, 2015). Regarding obesity-related barriers, literature shows body size, bodily pain, fatigue, injuries, discomfort, body complexes, embarrassment and humiliation when their bodies were publicly exposed, as the most prevalent barriers to PA (Beltrán-Carrillo et al., 2019; Toft & Uhrenfeldt, 2015; Zabatiero et al., 2018). Moreover, population with severe obesity use to describe their PA experiences as repeated unsuccessful attempts to lose weight (Zabatiero et al., 2018).

The complex phenomenon of severe obesity requires more qualitative research, especially when there are still few qualitative studies shedding light on the sociocultural and the psychological elements associated with the formation of severe obesity. Concretely, the aim of this qualitative study was to gain a deeper understanding of some elements which may influence sedentary lifestyles and unhealthy eating habits in individuals who lived with severe obesity during many years of their lives. Focusing on the subjective perspective of this population might help to find more efficient, sensitive and respectful strategies for severe obesity prevention/treatment.

## Method

This study followed a descriptive qualitative design and was underpinned by a “relativist ontology” and a “constructivist epistemology” (Sparkes & Smith, 2014), conceiving that social reality is subjectively perceived and humanly constructed, and assuming that generation of knowledge is not free of researchers’ values and previous knowledge. The concrete methodological details of this study are described in the following sections.

## Participants and procedure

The participants were ten post-bariatric patients (nine women and one man) aged between 31 and 59 years (*M* = 46.60, *SD* = 9.21). Some of their characteristics are described in Table I. Requirements for bariatric surgery were having a BMI greater than 40 kg/m^2^ or greater than 35 kg/m^2^ with associated comorbidity; being between 18 and 60 years old; having experienced previous failed attempts of sustained weight loss after endocrinology and nutritional counselling; and having no physical, medical or psychosocial contraindications. Bariatric patients’ perspective was very interesting for the aim of this study, because they had a previous long experience living with severe obesity and have lived several failed attempts to lose weight along their lives.

**Table I.** Characteristics of the Participants

Pseudonym	Gender	Age	BMI (1-month post-surgery)	Occupation	Marital status
Telma	Female	31	41.3	Cobbler	Single
Alice	Female	31	34.3	Hairdresser	Married
Pam	Female	45	36.9	Cobbler	Married
Emily	Female	49	35.6	Homemaker	Married
Susan	Female	50	37.6	Cleaner	Married
Lucy	Female	53	41.3	Cobbler	Divorced
Lezly	Female	54	39.8	Psychiatrist	Single
Sofie	Female	59	34.7	Homemaker	Married
Ronda	Female	45	41.3	Cook	Divorced
Andrew	Male	49	38.7	Taxi driver	Married

Note. BMI = Body Mass Index.

This qualitative study was part of a broader research project which employed a mixed method design to analyse the links between social environment and bariatric patients’ lifestyles. The project was approved by the ethical research board of the first author’s university. The participants were recruited from a Spanish hospital (University Hospital of Vinalopó, Elche) by a clinical psychologist during their preoperative visit. The first 10 participants who consulted the clinical psychologist were recruited because of all them were asked and agreed to participate.

Two in-depth semi-structured interviews were conducted with each participant and recorded to collect qualitative data. Both sets of interviews lasted between 40 and 60 minutes. One patient did not participate in the second interview, without giving any reason. The interviews were conducted by AM (initials of one co-author of this article) and took place in a quiet room at the first author’s research centre. The first interview was conducted 7 months after surgery. It included general questions to gather information about adherence to healthy habits (e.g., How were your habits regarding diet and physical activity before surgery?) and the experience of living with severe obesity (e.g., What has obesity meant in your live? How has obesity affected your life?). Interesting information was obtained from this first set of interviews about patients’ previous sedentary lifestyles, unhealthy habits and difficulties lived with obesity. However, we identified some knowledge gaps concerning the elements which influenced these habits, which we tried to solve adding new questions in a second interview carried out one year later (questions included: What were the main reasons that led you to obesity from your viewpoint? When and why did you start to gain weight? How was this process from normal or less weight to severe obesity? Do you think that your dietary habits led you to obesity? Why? Do you think that your physical inactivity led you to obesity? Why? Do you think that there were other elements [physical, psychological, social, environmental, etc.] which influenced your sedentary lifestyle or unhealthy eating habits? Have you ever tried to follow a diet? What diet? Did it work? Why? Have you ever tried to do exercise? Did you give it up? Why?).

Participants were informed about the study procedures and provided written consent. As the study was focused on information which was personal and private, or which contained evaluations of other people, the participants’ anonymity was protected using pseudonyms.

## Data analysis

Semi-structured interviews were transcribed and analysed with the support of the NVivo software, which was used to organize and classify data efficiently (Bazeley & Jackson, 2013). Qualitative data were analysed following a “content analysis” (Hsieh & Shannon, 2005) carried out in two phases. First, all transcriptions from the first interviews were read several times to become familiar with the data. Then, the fragments of text that captured key thoughts or concepts related to the purpose of the study were coded. Finally, these codes were inductively sorted into a system of interrelated categories and subcategories which provided meaning to the data in accordance to the study aim (e.g., The text fragment “I felt embarrassed about going to the pool because I was so fat” was coded as “Embarrassment about exposing their body in public”, which finally was a subcategory representing an element related to sedentary lifestyles). This first analysis was useful to identify knowledge gaps and new questions, which were used in the second batch of interviews (see Participants and procedure). In the second phase of data analysis, the information from the second interviews was also read and coded. These new codes were used to complete, refine and readjust the previous system of interrelated categories and subcategories. The final categorical system (Table II) was coherent for the researchers and sustained the findings presented in the results section.

**Table 2:** Elements Behind Participants’ Sedentary Lifestyles and Unhealthy Eating Habits

Categories	Subcategories
Sedentary lifestyles	Lack of time
	Economic barriers
	Low motivation
	Other leisure preferences
	Pain and poor physical condition
	Embarrassment about exposing their body in public
	Non-inclusive PA facilities for people with obesity
Unhealthy eating habits	Unhealthy diet and excessive eating
	• *Poor family eating education*
	• *Lack of time to prepare healthy food*
	Compulsive snacking and cravings
	• *Lack of self-control with food*
	• *Addiction to food*
	Emotional and comfort eating
	• *Family problems*
	• *Loss of a loved one*
	• *Arguments or disputes*
	• *Past traumatic event / Childhood sexual abuse*
	• *Low self-esteem, anxiety and depression*
Giving up diet and exercise after failed attempts to lose weight	Struggling with diets with high doses of sacrifice and very few results
	Anxiety, feelings of failure, guilt and rebellion
	Returning to an unhealthy diet and a sedentary lifestyle, rebound effects, regaining weight

*Note*. PA = Physical Activity.

AM led the analysis, whereas VB, AJ and DG played the role of “critical friends”, sharing ideas and reflections with their colleague to improve the quality and rigour of the coding/categorization process (Smith & McGannon, 2018). During a series of three meetings, AM presented the data analysis using diagrams, outlined the codes included in the different categories, and responded to the questions and suggestions of the critical friends. As part of the analysis, AM wrote the first draft of the article and the critical friends helped to improve its write-up until a final version was achieved.

## Findings and discussion

The elements behind participants’ sedentary lifestyles and unhealthy eating habits (see Table II) are described and discussed in the following sections.

### Sedentary lifestyles

A sedentary life was one of the most discussed conditions that participants believed contributed to their weight gain leading to obesity. Some participants commented that they barely made any physical efforts, and hardly did any exercise throughout their life history before surgery:

I have always been sedentary … I used the car to go everywhere, and I parked the car as near as possible to the place where I was going, walking as least as possible … No climbing stairs. I have always been touchy about climbing stairs … (Lezly; woman, 54 years old, second interview)

I did nothing. Moreover, I have to remain seated all day at work; after sitting down, I would switch to the sofa … The four things you do at home and that’s all. No PA, not even walking. My mom lives very close, and I would drive the car [to visit her] … (Pam; woman, 45 years old, second interview)

Lack of time is one of the most commented reasons participants claimed for their lack of exercise. Either because of taking care of their children, their studies or their jobs, participants hardly found any time to do exercise:

When I started to be obese at age 20, I started to do exercise, right? … I gave up exercise … because of the classes … I was studying medicine, and I didn’t have enough time, so I was leaving exercise aside … (Lezly; woman, 54 years old, second interview)

Apart from lack of time, one participant, Lucy, reported lack of economic resources as another reason for not exercising:

… I like the gym, but if I don’t work, I have no money, and if I work, when could I go? (Lucy; woman, 53 years old, first interview)

Participants also declared that they had very little interest in or motivation for PA and gave priority to any other behaviour instead of exercise:

On weekends, when I didn’t work, I went for a walk on the beach or I went cycling … It was alright at the beginning, but I lost interest very soon … I don’t know why. I started to give up and to get lazy. One day, because there was a football match on TV, another day, because we had a date with someone, and another day, because there was a TV series. I gave priority to anything except what I should do. (Andrew; man, 49 years old, second interview)

Moreover, a vicious circle between sedentary lifestyle and obesity occurred, as weight gain was associated with inactivity (and vice versa) through the progressive deterioration of physical condition. One participant, Lezly, clearly explained this process in the following quotation..

… there is a vicious circle between obesity and lack of exercise. One thing leads to the other. With excessive weight … your legs hurt, you don’t have energy to walk or do exercise because you get exhausted and you don’t feel good. And the lack of exercise promotes weight gain, as you burn fewer calories than you eat. (Lezly; woman, 54 years old, second interview)

Finally, participants admitted that they were very embarrassed and concerned about their body shape when having to do PA in public (*Andrew: … I felt embarrassed in a spinning class of a normal gym; Sofie: … I felt embarrassed about going to the pool because I was so fat)*. Moreover, if they finally got involved in PA programmes, they felt biased against and marginalized in gyms because they perceived these facilities as non-inclusive places and saw themselves very different from the sort of people who attended these facilities. They also felt that the staff and instructors did not look after them or their specific needs. Therefore, they finally stopped going:

… if you go to any gym, they don’t pay any attention to you … there, you see a muscular guy, like the rest of guys attending these places, and he tells you, “ten minutes on the treadmill”, and he goes away. They give you a notebook … I didn’t know how it worked, and they explained four exercises to me and ignored me. So, then, you quit going. (Lezly; woman, 54 years old, first interview)

The association between sedentary behaviours and obesity development has been widely reported (Brogan & Hevey, 2009; Romieu et al., 2017). Regarding our participants’ explanations for their inactivity, qualitative literature coincides with them, as lack of time, low financial resources, lack of motivation and laziness have been found to be the most commented barriers to PA, not only by population with obesity but also by general population (Napolitano et al., 2011; Toft & Uhrenfeldt, 2015; Zabatiero et al., 2018). Concerning obesity-related barriers, previous studies have reported bodily pain, fatigue, body complexes and embarrassment when their bodies were publicly exposed (Beltrán-Carrillo et al., 2019; Toft & Uhrenfeldt, 2015; Zabatiero et al., 2018). This last barrier is related to the current social obesity discourse, which promotes society’s stereotype of obesity as a disease/disability resulting from the individual’s laziness and lack of self-control or willpower (Gard, 2011; Jiménez-Loaisa et al., 2020; Puhl & Heuer, 2010). This individualist discourse places the responsibility for health firmly on the individual and conceives the slim, fit, and muscular body as a symbol of health. In this way, this obesity discourse promotes stigmatization, discrimination, and negative attitudes towards people with obesity in many social contexts, including exercise contexts.

### Unhealthy eating habits

#### Unhealthy diet and excessive eating

Participants admitted through their comments that they had followed an unhealthy diet their whole life. They used to consume products rich in fat and sugar and eat large amounts of food without any control:

… I ate very poorly. My Mom would make an omelette and I could eat the whole omelette. I loved fried food, I never had enough. I could eat a whole baguette deeping in the fried oil. (Pam; woman, 45 years old, second interview)

… I ate what I shouldn’t and I had no control. I ate what I felt like eating. I would finish my breakfast and then I would say, ‘I am ready for a stew’. And I would eat it with the meatballs, beans … And after that, if I felt like eating pie, I would eat a piece of pie. (Lucy; woman, 53 years old, second interview)

Food intake that exceeds energy expenditure has been reported to be the main driver of weight gain, ahead of other elements included in the equation such as lack of physical activity (Romieu et al., 2017). Along with this evidence, the high availability of obesogenic processed food in developed industrialized societies should also be highlighted (Basu et al., 2013). High consumption of certain food types such as French fries, sweets, meat, cheese, butter, high-fat snacks, fried foods and desserts has been negatively associated with weight maintenance (Elfhag & Rossner, 2005).

Many participants highlighted the poor family eating education they received during their childhood, as they followed hyper-caloric and fatty diets (*Alice: … the diet at home was always based on pork, fatty meat and potatoes; Telma: … fried food … I got used to that because, at home, we ate fried food every night*). One participant even related how her family associated health with overweight, influenced by a “healthy-chubby” notion, which was common several decades ago in Spain among people who had lived social periods of certain food scarcity:

My mother didn’t care whether the food promoted gaining weight. “If you like it, you eat it”. She cooked every kind of food we liked. My mother associated being chubby with being healthy. We are five sisters, and all of us are chubby. (Pam; woman, 45 years old, second interview)

In this sense, Braun et al. (2014) found in a qualitative study that obesity was generally framed as a problem primarily located within the family and not in the broader environment. Moreover, the lack of time to prepare the food appropriately and to eat calmly is another element that participants reported as a reason for consuming junk food or high-calorie snacks:

… when you work, you don’t cook the same; you just grab a bag of crisps, a donut … you stave off the hunger with this stuff … (Emily; woman, 49 years old, second interview)

Regarding this issue, a previous study found associations between current employment and frequent fast-food intake, probably due to hectic work schedules and workplace environments, which may predispose individuals to consume fast-food products (Salisbury et al., 2011). Moreover, the prevalence of fast-food consumption has been strongly associated with obesity (Basu et al., 2013; De Vogli et al., 2014).

#### Compulsive snacking and cravings

Many participants acknowledged that they had no control over snacking on a lot of hyper-caloric junk food over the day. This difficulty to manage cravings has been shown to be counter-productive to weight maintenance (Elfhag & Rossner, 2005) and associated with obesity (Nuru & Mamang, 2015). Food was always on participants’ mind and strongly associated with their daily routines:

I would be working and eating crisps, with a 2-litre coke ready, some peanuts … eating all day. (Pam; woman, 45 years old, first interview)

… I was watching TV and thinking, “what can I eat? … I got up and ate a banana, after that, I prepared a glass of milk with biscuits … (Telma; woman, 31 years old, second interview)

… in the afternoons it was extreme. Going to the kitchen, opening the fridge or the cupboard, getting something … back to the sofa. When I finished it, after half an hour, I had to go to the kitchen to grab something else. I ate it, and in half an hour, all over again! I had to go to the kitchen to grab something else. (Emily; woman, 49 years old, first interview)

It could be said that participants had a psychological addiction to food (Ueland et al., 2019), as food was totally associated with their daily routines, and it was a constant thought in their minds, even describing night eating episodes in some cases. This repeated impulse for immediate satisfaction through food is related to a personality trait associated with some types of psychogenic obesity as well as with drug addiction (Elfhag & Rossner, 2005). In fact, an addictive personality has been found to act distally on over-eating and, therefore, on obesity (Brogan & Hevey, 2009).

#### Emotional and comfort eating

Participants admitted using food to calm anxiety initiated by any problem. A common characteristic in people struggling with weight is that they tend to eat in response to stressful or negative life events, using food to regulate mood and negative emotions (Brogan & Hevey, 2009; Elfhag & Rossner, 2005; Puhl & Heuer, 2010; Ueland et al., 2019). This behaviour has been conceptualized as “comfort eating” by Brogan and Hevey (2009), and places people in a perpetual cycle of mood disturbance, overeating and weight gain. Several quotations reflected this sequence:

I was very nervous … because of family problems … My son was a drug addict, and I started eating. I became more and more overweight … (Sofie; woman, 59 years old, second interview)

… my father passed away. I started eating and crying, and I put on a lot of weight … Any time I’ve had a problem, I’ve always turned to eating. (Pam; woman, 45 years old, second interview)

… I had a problem or argued with someone and … by eating … I felt comfortable, and it was like the problem disappeared. (Telma; woman, 31 years old, second interview)

Lastly, one participant (Emily) related how a traumatic experience of sexual abuse in her childhood was the trigger of her obesity problem. This participant described that, after getting married, this traumatic past event of her childhood was rekindled, causing her a lot of anxiety, which she tried to cope with by eating compulsively:

… I had a trouble [sexual abuse during her childhood] … so much anxiety … you cope with that anxiety, apart from crying … by eating, and keeping your mind busy by eating when you aren’t crying … (Emily; woman, 49 years old, second interview)

I started gaining weight when I got married. I got married when I was 21, and from this point on, I put on almost 20 kilograms. I weighed 50 kg when I got married and then I went up to 70 kg. (Emily; woman, 49 years old, second interview)

Literature has found that childhood negative experiences could be associated with severe obesity (Ueland et al., 2019) and traumatic events act distally on over-eating and comfort eating (Brogan & Hevey, 2009). Relatively few studies have examined the relationship between childhood sexual abuse and adult obesity (Gustafson & Sarwer, 2004; Smith et al., 2010). However, these studies have identified poor self-esteem, poor body image, impulsive behaviour and depression as possible consequences of child abuse, and these variables are common predictors of binge eating or compulsive eating. These eating behaviours are probably adopted to manage the depression related to child sexual abuse, and could play an adaptive and “de-sexualizing” function to protect against further abuse.

In summary, participants’ problematic eating behaviours were frequently related to their daily troubles or to relevant negative events in their lives, which produced low self-esteem, anxiety or depression:

*…* when I’ve felt down, I’ve always reacted by eating … (Pam; woman, 45 years old, second interview)

… my obesity problem was due to anxiety and depression … I think it was because of my depression. (Lucy; woman, 53 years old, second interview)

Other studies have shown that there is an overlap between obesity and mood disorders like depression (Chauvet-Gelinier et al., 2019; Luppino et al., 2010). Depression increases the risk of developing obesity (Luppino et al., 2010), much more so in case of disadvantaged groups, including women, because they tend to have access to fewer stress-buffering resources (Sutin & Zonderman, 2012). In addition, depression is associated with reduced PA, which may also lead to weight gain (Delgado-Floody et al., 2021).

### Giving up diet and exercise after failed attempts to lose weight

Participants described how diets to lose or maintain weight had been part of their life history. They remembered struggling to follow diets, needing high doses of sacrifice and self-control to cope with hunger and to avoid those high-calorie products they loved and used to eat. Dieting was an important source of stress and anxiety for many participants, who lived under rigid self-control throughout their lives to control their weight. This stress reached the highest level when they did not perceive weight loss, turning to food to cope with discomfort and giving up exercise if they were doing it:

It’s tiring. A sheet with instructions for lunch, snack, breakfast and dinner. And the same thing the following week. When I went for the monthly visit, the doctor asked me, “How have you only lost five hundred grams?” I followed all the instructions written on the paper, but … you realize you are making a big sacrifice, but you don’t lose weight … you get more anxious and more nervous, and you turn to the same old thing … to eating because of your nerves. (Emily; woman, 49 years old, second interview)

… one day, I said … I’m going to go on a diet … and … I signed up in a gym. I started and when I realized I was getting bogged down again, I quit the diet and I gave up the gym, and I regained the weight and even gained twice the weight. After two months … I went on a diet again. The same thing happened … I have been living like that my whole life. (Telma; woman, 31 years old, second interview)

I have been on a diet for half my life. You get tired at the end … you always eat the same stuff and … there comes a moment … ‘Fuck, I feel like going to a terrace and having an ice-cream’, ‘Why can’t I go to a terrace and have an ice-cream?’ But always controlling the food issue. (Alice; woman, 31 years old, first interview)

Previous studies have found that individuals experience their battle with obesity as something that has always been present, as a lifelong battle of short-term weight loss and longer-term weight gain (Braun et al., 2014; Elfhag & Rossner, 2005; Greener et al., 2010; Haga et al., 2020; Toft & Uhrenfeldt, 2015). In fact, only the 5% of people who attempt to lose large amounts of weight experience long-term success (Ikeda et al., 2005). This life of continuous struggle, trying to control weight and failing, had important consequences on participants. For instance, participants struggled inside a vicious circle of “distress-eating-distress”, and felt guilty about that:

The worst thing for me has been anxiety. I lost control … My escape was food … a piece of cheese, some salami, now a cookie, chocolate … Eating, eating … After that, sometimes, I felt angry … but, as I had already eaten … (Lucy; woman, 53 years old, second interview)

This finding is in line with other studies showing how most people with obesity struggle in vain to lose weight and blame themselves for relapses, experiencing failure, overall discouragement and impaired self-confidence (Elfhag & Rossner, 2005; Greener et al., 2010). Some participants even reacted to this distress by neglecting their health through binge eating/snacking, or giving up exercise:

They told me to walk, I lay down on the couch. “Don’t eat that”, and I ate it. I rebelled against them. I don’t know what happened … My head didn’t react … Instead of taking care of myself, I wanted to destroy myself. (Lucy; woman, 53 years old, second interview)

According to Kopetz and Orehek (2015), adults engage in a variety of self-defeating behaviours as a result of breakdowns of self-regulations. Participants dieting through high doses of self-control, when failing to diet, can react by eating large quantities of food without control (binge eating) and believing they are unable to control their weight (Elfhag & Rossner, 2005). This situation results in an abandonment of weight maintenance behaviours (Zabatiero et al., 2018).

## Conclusions and recommendations

The present research has identified several elements associated with sedentary lifestyles and unhealthy eating habits in a group of post-bariatric surgery patients who had lived with severe obesity during many years of their lives. These findings are in line with existing research, as illustrated in the previous section.

With regard to sedentary lifestyles, participants perceived many barriers to PA. Some of them were not specifically related to obesity, such as lack of time, limited financial resources and lack of motivation. To overcome lack of time and promote active lifestyles, a work schedule more compatible with personal life and leisure would be desirable. Promoting PA at work could also be interesting, offering active breaks throughout the working day and exercise areas in the company’s facilities. Economic barriers could be reduced by implementing health education programmes for patients with obesity, including content to promote healthy exercise on their own without the need of expensive equipment. Another measure could be the offer, by the public health system, of low-cost PA programmes supervised by doctors and run by sport science professionals. In this regard, previous studies indicate that investment in the promotion of PA can reduce the economic cost of health systems (Ding et al., 2016). These health-education and PA programmes should include motivational training so that patients are motivated to be active in the long term. The professionals in charge of these programmes should also be trained in the special needs and characteristics of this population. The Healthy Life Centres, as part of primary health care services in Norway, represent a valuable example of initiatives from the health system to promote healthy habits. These centres were established at the early 2000s and interesting research has been carried out about them (e.g., Gjertsen et al., 2021; Sevild et al., 2020).

Other barriers to PA were associated with obesity, such as pain and poor physical condition, embarrassment about exposing their body in public, and the existence of non-inclusive PA facilities for people with obesity. These patients should know that doing exercise is crucial to break the vicious circle of “poor physical condition-inactivity-worse physical condition”. This argument should be included in the messages disseminated in the interventions for the promotion of PA, although the target population should know that they need to be advised by qualified professionals to do individualized, correct and healthy exercise. Moreover, interventions for the promotion of PA should be focused on the promotion of health, wellbeing and healthy behaviours, avoiding an excessive weight-management approach (Jiménez-Loaisa et al., 2015). This could be helpful to reduce patients’ concerns about their body shape, reinforce their self-esteem and reduce their embarrassment of doing exercise in front of others. It would also be necessary to guarantee inclusive PA contexts in which participants can feel respected by the instructors in charge of the activities and other exercisers. PA facilities should also possess specific designs and physical structures to safeguard patients’ privacy if they have body-complexes and do not want to be observed by others (Jiménez-Loaisa et al., 2015). For instance, the availability of individual showers or swimming pools closed to external observers would be desirable.

Regarding their unhealthy eating habits, participants admitted to have followed an inappropriate diet for many years of their lives and to have eaten excessively and compulsive. However, as in the case of sedentary lifestyles, the interesting contribution of this article lies in shedding light on the elements that could be related to these eating behaviours.

In this study, family food education has been identified as an important element related to the unhealthy eating behaviours of the participants. Strategies for the prevention of obesity should pay special attention to this issue. Health centres should promote health education courses among families with special lack of knowledge about healthy diet. Schools can also play a very important role in the prevention of obesity and unhealthy diet in childhood. Another element was the lack of time to prepare healthy food. A readjustment of labour schedules to make them more compatible with family life could palliate this problem. In addition, the food industry should invest in a more extensive availability of healthy food.

Our findings also shed light on the diverse problems and negative life events which can underlie the unhealthy eating behaviours of people with severe obesity (family problems, loss of a loved one, arguments or disputes, childhood sexual abuse). Early psychological intervention to treat the psychological problems (low self-esteem, anxiety, and depression) which can lead to emotional and comfort eating should be part of the strategies for obesity prevention. In fact, Eik-Nes et al. (2021) showed that psychoeducational group interventions that address the underlying psychological mechanisms of binge eating and obesity can be useful to improve health-related quality of life and reduce binge eating.

A lifelong struggle with diets and failed attempts to lose weight were also associated with the PA and eating behaviours of the participants. This long, unsuccessful process of control and restriction contributed to their distress and deliberate abandonment of weight maintenance behaviours. In this sense, people with severe obesity should understand that specific changes in diet and PA patterns for concrete periods of time make no sense. A real change of lifestyle is what is needed for the treatment and prevention of obesity. These changes in diet and PA could be more successful if they were more focused on improving health and wellbeing, and less focused on losing weight. Likewise, these lifestyle changes could be more successful if they are concurrent. Patients with obesity who participate in PA could reduce their weight with less restrictions in the energy intake (Moya et al., 2014), palliate their emotional problems and improve mental health (Jiménez-Loaisa et al., 2015). The support of health professionals, couples, family, friends, and other patients with severe obesity could be of vital importance to achieve these lifestyle changes and reduce the risk of giving up exercise or turning to comfort eating. In this regard, it would be interesting to train health professionals in behaviour change techniques, so that they can motivate patients during obesity treatment, instead of promoting feelings of failure as some patients stated.

Participants described that they were physically inactive and practiced unhealthy eating behaviours for many years throughout their lives, reaching a condition of severe obesity. However, although individual responsibility concerning the aetiology of severe obesity is evident, our work is aligned with initiatives such as Health At Every Size (HAES; see https://haescommunity.com/), which recognize that health outcomes are driven primarily by social, economic and environmental conditions that require a social and political response. Concretely, we have described the diverse educational, psychological and social elements, as well as the traumatic life events, which can be associated with the emergence and maintenance of these undesirable behaviours. This information may be helpful to reveal some elements related to the origin of severe obesity, and to design prevention/treatment strategies from a more holistic, sensitive, and respectful perspective. This comprehensive view is necessary in a society which tends to consider people with severe obesity as lazy, irresponsible, and guilty of their health problems (Gard, 2011; Jiménez-Loaisa et al., 2020).

## Strengths and limitations of the study

Some methodological considerations enhanced the trustworthiness or rigour of our study. Two in-depth semi-structured interviews were carried out with each participant, both of them separated one year of time, with a process of data analysis after each set of interviews. This prolonged engagement in the process of data collection and analysis let us identify knowledge gaps related to the study and collect new data to address them. This methodological aspect, which can favour rigour in qualitative research, is not possible in cross-sectional studies (Shenton, 2004; Strauss, 1987). In addition, the supervision and active collaboration of the “critical friends” were useful to increase the quality of data analysis and the writing of the article (Shenton, 2004; Smith & McGannon, 2018).

Several limitations must also be recognized. The sample of our study was reduced, and our findings cannot be generalized to other populations with different profiles and from different contexts. Also, most of the participants were women and they can life obesity in a different way than men. The ideal body for women has been associated with beauty, youth, sensuality and thinness, and the social pressure to have an ideal body has historically been higher for women than men (Jiménez-Loaisa et al., 2020; Toro, 2003). Moreover, our participants were bariatric patients and probably lived their obesity in a different way than people with severe obesity that decided not to undergo bariatric surgery or even lose weight. Nevertheless, from our point of view, an in depth understanding of this subsample represents an interesting contribution to the literature on this topic. Academics and professionals concerned with the health and wellbeing of patients with severe obesity could find useful and transferable information in this article.

## References

[cit0001] Basu, S., McKee, M., Galea, G., & Stuckler, D. (2013). Relationship of soft drink consumption to global overweight, obesity and diabetes: A cross-national analysis of 75 countries. *American Journal of Public Health*, 103(11), 2071–11. 10.2105/AJPH.2012.300974 23488503 PMC3828681

[cit0002] Bazeley, P., & Jackson, K. (2013). *Qualitative data analysis with NVivo*. Sage.

[cit0003] Beltrán-Carrillo, V. J., Jiménez-Loaisa, A., Jennings, G., González-Cutre, D., Navarro-Espejo, N., & Cervelló, E. (2019). Exploring the socio-ecological factors behind the (in)active lifestyles of Spanish post-bariatric surgery patients. *International Journal of Qualitative Studies on Health and Well-Being*, 14(1), Article 1626180. 10.1080/17482631.2019.1626180 31187702 PMC6566659

[cit0004] Braun, M., Schell, J., Siegfried, W., Müller, M. J., & Ried, J. (2014). Re-entering obesity prevention: A qualitative-empirical inquiry into the subjective etiology of extreme obese adolescents. *BMC Public Health*, 14(1), Article 977. 10.1186/1471-2458-14-977 PMC4177709 25239081

[cit0005] Brogan, A., & Hevey, D. (2009). The structure of the causal attribution belief network of patients with obesity. *British Journal of Health Psychology*, 14(1), 35–48. 10.1348/135910708X292788 18426688

[cit0006] Brownell, K. D., & Horgen, K. B. (2004). *Food fight: The inside story of the food industry, America’s obesity crisis, and what we can do about it*. McGraw-Hill Education.

[cit0007] Chauvet-Gelinier, J. C., Roussot, A., Cottenet, J., Brindisi, M. C., Petit, J. M., Bonin, B., Vergès, B., & Quantin, C. (2019). Depression and obesity, data from a national administrative database study: Geographic evidence for an epidemiological overlap. *PloS One*, 14(1), Article e0210507. 10.1371/journal.pone.0210507 PMC6324832 30620759

[cit0008] De Vogli, R., Kouvonen, A., & Gimeno, D. (2014). The influence of market deregulation on fast food consumption and body mass index: A cross-national time series analysis. *Bulletin of the World Health Organization*, 92(2), 99–107. 10.2471/BLT.13.120287 24623903 PMC3949530

[cit0009] Delgado-Floody, P., Guzmán-Guzmán, I. P., Caamaño-Navarrete, F., Jerez-Mayorga, D., Zulic-Agramunt, C., & Cofré-Lizama, A. (2021). Depression is associated with lower levels of physical activity, body image dissatisfaction, and obesity in Chilean preadolescents. *Psychology, Health & Medicine*, 26(4), 518–531. 10.1080/13548506.2020.1817958 32885991

[cit0010] Ding, D., Lawson, K. D., Kolbe-Alexander, T. L., Finkelstein, E. A., Katzmarzyk, P. T., van Mechelen, W., & Pratt, M. (2016). The economic burden of physical inactivity: A global analysis of major non-communicable diseases. *The Lancet*, 388(10051), 1311–1324. 10.1016/S0140-6736(16)30383-X 27475266

[cit0011] Distel, L. M. L., Egbert, A. H., Bohnert, A. M., & Santiago, C. D. (2019). Chronic stress and food insecurity: Examining key environmental family factors related to body mass index among low-income Mexican-origin youth. *Family & Community Health*, 42(3), 213–220. 10.1097/FCH.0000000000000228 31107732

[cit0012] Eik-Nes, T. T., Vrabel, K., Raman, J., Clark, M. R., & Berg, K. H. (2021). A group intervention for individuals with obesity and comorbid binge eating disorder: Results from a feasibility study. *Frontiers in Endocrinology*, 12, Article 738856. 10.3389/fendo.2021.738856 34803910 PMC8597950

[cit0013] Elfhag, K., & Rossner, S. (2005). Who succeeds in maintaining weight loss? A conceptual review of factors associated with weight loss maintenance and weight regain. *Obesity Reviews*, 6(1), 67–85. 10.1111/j.1467-789X.2005.00170.x 15655039

[cit0014] Gard, M. (2011). Truth, belief and the cultural politics of obesity scholarship and public health policy. *Critical Public Health*, 21(1), 37–48. 10.1080/09581596.2010.529421

[cit0015] Gjertsen, T. I., Helvik, A. S., & Følling, I. S. (2021). Previous life experiences and social relations affecting individuals wish for support when establishing healthy habits – A qualitative study of Norwegian Healthy Life Centre participants. *BMC Public Health*, 21(1), Article 1315. 10.1186/s12889-021-11374-8 34225666 PMC8256571

[cit0016] Greener, J., Douglas, F., & van Teijlingen, E. (2010). More of the same? Conflicting perspectives of obesity causation and intervention amongst overweight people, health professionals and policy makers. *Social Science & Medicine*, 70(7), 1042–1049. 10.1016/j.socscimed.2009.11.017 20106576

[cit0017] Gustafson, T. B., & Sarwer, D. B. (2004). Childhood sexual abuse and obesity. *Obesity Reviews*, 5(3), 129–135. 10.1111/j.1467-789X.2004.00145.x 15245381

[cit0018] Haga, B. M., Furnes, B., Dysvik, E., & Ueland, V. (2020). Putting life on hold: Lived experiences of people with obesity. *Scandinavian Journal of Caring Sciences*, 34(2), 514–523. 10.1111/scs.12756 31638725

[cit0019] Hernáez, A., Zomeño, M. D., Dégano, I. R., Pérez-Fernández, S., Goday, A., Vila, J., Civeira, F., Moure, R., & Marrugat, J. (2019). Excess weight in Spain: Current situation, projections for 2030, and estimated direct extra cost for the Spanish health system. *Revista Española de Cardiología*, 72(11), 916–924. 10.1016/j.rec.2018.10.010 30473259

[cit0020] Hsieh, H., & Shannon, S. E. (2005). Three approaches to qualitative content analysis. *Qualitative Health Research*, 15(9), 1277–1288. 10.1177/1049732305276687 16204405

[cit0021] Ikeda, J., Amy, N. K., Ernsberger, P., Gaesser, G. A., Berg, F. M., Clark, C. A., Parham, E. S., & Peters, P. (2005). The national weight control registry: A critique. *Journal of Nutrition Education and Behavior*, 37(4), 203–205. 10.1016/S1499-4046(06)60247-9 16029691

[cit0022] Jiménez-Loaisa, A., Beltrán-Carrillo, V. J., González-Cutre, D., & Cervelló, E. (2015). Psychosocial effects of surgery and physical activity in bariatric patients: A systematic review. *European Journal of Human Movement*, 35, 12–33. https://www.eurjhm.com/index.php/eurjhm/article/view/360

[cit0023] Jiménez-Loaisa, A., Beltrán-Carrillo, V. J., González-Cutre, D., & Jennings, G. (2020). Healthism and the experiences of social, healthcare and self-stigma of women with higher-weight. *Social Theory and Health*, 18(4), 410–424. 10.1057/s41285-019-00118-9

[cit0024] Konttinen, H., Sarlio-Lähteenkorva, S., Silventoinen, K., Männistö, S., & Haukkala, A. (2013). Socio-economic disparities in the consumption of vegetables, fruit and energy-dense foods: The role of motive priorities. *Public Health Nutrition*, 16(5), 873–882. 10.1017/S1368980012003540 22857602 PMC10271369

[cit0025] Kopetz, C., & Orehek, E. (2015). When the end justifies the means: Self-defeating behaviors as “rational” and “successful” self-regulation. *Current Directions in Psychological Science*, 24(5), 386–391. 10.1177/0963721415589329

[cit0026] Luppino, F. S., de Wit, L. M., Bouvy, P. F., Stijnen, T., Cuijpers, P., Penninx, B. W. J. H., & Zitman, F. G. (2010). Overweight, obesity, and depression. A systematic review and meta-analysis of longitudinal studies. *Archives of General Psychiatry*, 67(3), 220–229. 10.1001/archgenpsychiatry.2010.2 20194822

[cit0027] McCuen-Wurst, C., Ruggieri, M., & Allison, K. C. (2018). Disordered eating and obesity: Associations between binge eating-disorder, night-eating syndrome, and weight-related co-morbidities. *Annals of the New York Academy of Sciences*, 1411(1), 96–105. 10.1111/nyas.13467 29044551 PMC5788730

[cit0028] Moya, M., Hernández, A., Sarabia, J. M., Sánchez-Martos, M. A., Hernández-Davó, J. L., López-Grueso, R., Aracil, A., Pastor, D., & Fernández-Fernández, J. (2014). Bariatric surgery, weight loss and the role of physical activity: A systematic review. *European Journal of Human Movement*, 32, 145–160. https://www.eurjhm.com/index.php/eurjhm/article/view/323

[cit0029] Napolitano, M. A., Papandonatos, G. D., Borradaile, K. E., Whiteley, J. A., & Marcus, B. H. (2011). Effects of weight status and barriers on physical activity adoption among previously inactive women. *Obesity*, 19(11), 2183–2189. 10.1038/oby.2011.87 21512514

[cit0030] Nuru, H., & Mamang, F. (2015). Association between snacking and obesity in children: A review. *International Journal of Community Medicine and Public Health*, 2(3), 196–200. 10.18203/2394-6040.ijcmph20150472

[cit0031] Puhl, R. M., & Heuer, C. A. (2010). Obesity stigma: Important considerations for public health. *American Journal of Public Health*, 100(6), 1019–1028. 10.2105/AJPH.2009.159491 20075322 PMC2866597

[cit0032] Puhl, R. M., Lessard, L. M., Larson, N., Eisenberg, M. E., & Neumark-Stzainer, D. (2020). Weight stigma as a predictor of distress and maladaptive eating behaviors during COVID-19: Longitudinal findings from the EAT study. *Annals of Behavioral Medicine*, 54(10), 738–746. 10.1093/abm/kaaa077 32909031 PMC7499477

[cit0033] Rolls, B. J., Roe, L. S., Halverson, K. H., & Meengs, J. S. (2007). Using a smaller plate did not reduce energy intake at meals. *Appetite*, 49(3), 652–660. 10.1016/j.appet.2007.04.005 17540474 PMC2129126

[cit0034] Romieu, I., Dossus, L., Barquera, S., Blottière, H. M., Franks, P. W., Gunter, M., Hwalla, N., Hursting, S. D., Leitzmann, M., Margetts, B., Nishida, C., Potischman, N., Seidell, J., Stepien, M., Wang, Y., Westerterp, K., Winichagoon, P., Wiseman, M., & Willett, W. C. (2017). Energy balance and obesity: What are the main drivers? *Cancer Causes & Control: CCC*, 28(3), 247–258. 10.1007/s10552-017-0869-z 28210884 PMC5325830

[cit0035] Ruhlman, M. (2017). *Grocery: The buying and selling of food in America*. Abrams Press.

[cit0036] Salisbury, A. C., Chan, P. S., Gosch, K. L., Buchanan, D. M., & Spertus, J. A. (2011). Patterns and predictors of fast food consumption after acute myocardial infarction. *American Journal of Cardiology*, 107(8), 1105–1110. 10.1016/j.amjcard.2010.12.005 PMC3070863 21306695

[cit0037] Scott, K. A., Melhorn, S. J., & Sakai, R. R. (2012). Effects of chronic social stress on obesity. *Current Obesity Reports*, 1(1), 16–25. 10.1007/s13679-011-0006-3 22943039 PMC3428710

[cit0038] Sevild, C. H., Niemiec, C. P., Bru, L. E., Dyrstad, S. M., & Husebø, A. M. L. (2020). Initiation and maintenance of lifestyle changes among participants in a healthy life centre: A qualitative study. *BMC Public Health*, 20(1), Article 1006. 10.1186/s12889-020-09111-8 PMC7318496 32586299

[cit0039] Shenton, A. K. (2004). Strategies for ensuring trustworthiness in qualitative research projects. *Education for Information*, 22(2), 63–75. 10.3233/EFI-2004-22201

[cit0040] Smith, H. A., Markovic, N., Danielson, M. E., Matthews, Youk, A., Talbott, E. O., Larkby, C., Hughes, T. (2010). Sexual abuse, sexual orientation, and obesity in women. *Journal of Women’s Health*, 19(8), 1525–1532. 10.1089/jwh.2009.1763 PMC2941402 20524896

[cit0041] Smith, B., & McGannon, K. R. (2018). Developing rigor in qualitative research: Problems and opportunities within sport and exercise psychology. *International Review of Sport and Exercise Psychology*, 11(1), 101–121. 10.1080/1750984X.2017.1317357

[cit0042] Sparkes, A. C., & Smith, B. (2014). *Qualitative research methods in sport, exercise and health: From process to product*. Routledge/Taylor & Francis Group.

[cit0043] Strauss, A. (1987). *Qualitative analysis for social scientists*. Cambridge University Press.

[cit0044] Sutin, A., & Zonderman, A. (2012). Depressive symptoms are associated with weight gain among women. *Psychological Medicine*, 42(11), 2351–2360. 10.1017/S0033291712000566 22475128 PMC3439550

[cit0045] Toft, B. S., & Uhrenfeldt, L. (2015). The lived experiences of being physically active when morbidly obese: A qualitative systematic review. *International Journal of Qualitative Studies on Health and Well-Being*, 10(1), Article 28577. 10.3402/qhw.v10.28577 PMC4580712 26400462

[cit0046] Toro, J. (2003). *El cuerpo como delito. Anorexia, bulimia, cultura y sociedad [The body as crime. Anorexia, bulimia, culture and society]*. Ariel.

[cit0047] Ueland, V., Furnes, B., Dysvik, E., & Rørtveit, K. (2019). Living with obesity — Existential experiences. *International Journal of Qualitative Studies on Health and Well-Being*, 14(1), Article 1651171. 10.1080/17482631.2019.1651171 PMC6713124 31411129

[cit0048] Ward, Z. J., Bleich, S. N., Cradock, A. L., Barrett, J. L., Giles, C. M., Flax, C., Long, M. W., & Gortmaker, S. L. (2019). Projected U.S. state-level prevalence of adult obesity and severe obesity. *The New England Journal of Medicine*, 381(25), 2440–2450. 10.1056/NEJMsa1909301 31851800

[cit0049] World Health Organization (2021, June 9). *Obesity and overweight*. https://www.who.int/news-room/fact-sheets/detail/obesity-and-overweight

[cit0050] Zabatiero, J., Smith, A., Hill, K., Hamdorf, J. M., Taylor, S. F., Hagger, M. S., & Gucciardi, D. F. (2018). Do factors related to participation in physical activity change following restrictive bariatric surgery? A qualitative study. *Obesity Research & Clinical Practice*, 12(3), 307–316. 10.1016/j.orcp.2017.11.001 29150223

